# LncRNA PVT1 regulates biological function of osteoarthritis cells by regulating miR-497/AKT3 axis

**DOI:** 10.1097/MD.0000000000031725

**Published:** 2022-11-11

**Authors:** Jinming Xu, Xiang Fang, Ling Qin, Qiang Wu, Xinli Zhan

**Affiliations:** a Department of Spine and Osteopathy Ward, The First Affiliated Hospital of Guangxi Medical University, Nanning, China; b Department of Orthopedics, Yuebei People’s Hospital Affiliated to the Medical College of Shantou University, Shaoguan, China.

**Keywords:** AKT3, chondrocyte, miR-497, osteoarthritis, PVT1

## Abstract

Growing evidence indicates that lncRNAs are involved in the progression of several diseases, including osteoarthritis (OA). However, the role of the lncRNA PVT1 in OA is still unclear. The present study was aimed at exploring the impact of PVT1 on OA progression, along with potential underlying mechanisms. PVT1 expression levels in articular cartilage tissue of OA patients and non-OA patients were evaluated. To assess the proliferation and apoptosis of chondrocytes subject to treatment, PVT1, miR-497, and AKT3 were either knocked down or upregulated in IL-1β-induced chondrocytes. The variables detected were changes in levels of AKT3 and extracellular matrix (ECM)-related factors (including aggrecan, collagen Type II, and MMP-9). Elevated PVT1 levels were found in cartilage tissue of OA patients and IL-1β-induced chondrocytes. It was also observed that PVT1 knockdown and miR-497 upregulation led to enhanced cell proliferation and suppressed apoptosis. In addition, a decrease in aggrecan and collagen type II levels and an increase in MMP-9 levels were observed in IL-1β-induced chondrocytes. A dual luciferase reporter assay was performed to identify the factors that interacted with miR-497, PVT1, and AKT3. It was observed through rescue experiments that enhancing AKT3 expression or knocking down miR-497 could reverse the impacts of PVT1 knockdown in IL-1β-induced chondrocytes. An upregulation of PVT1 is observed in OA patients. On the other hand, PVT1 knockdown can decrease the effects of IL-1β on the proliferation, apoptosis, and expression of ECM-related proteins of chondrocytes through the regulation of the miR-497/AKT3 axis. PVT1 levels are elevated in the cartilage tissue of OA patients and IL-1β-induced chondrocytes. PVT1 knockdown alleviates the effects of IL-1β treatment on the proliferation and apoptosis of chondrocytes and ECM degradation in chondrocytes by regulating the miR-497/AKT3 axis.

## 1. Introduction

Osteoarthritis (OA), the most common degenerative disease of joints, is characterized by articular cartilage degeneration, subchondral osteosclerosis, and osteophyte formation.^[1]^ The main symptoms of OA include joint pain, joint stiffness, and decreased range of motion. These manifestations seriously affect the quality of life of OA patients.^[2]^ At present, OA is considered an incurable disease. The patients are mainly treated symptomatically in order to improve joint function and provide pain relief.^[3]^ Therefore, the potential mechanisms underlying OA pathogenesis need to be elucidated urgently in order to find promising therapeutic targets.

Long-chain non-coding RNAs (lncRNAs) are a group of non-coding RNAs (ncRNAs) with a length of more than 200 nucleotides. They have been shown to be involved in and regulate numerous biological processes, such as cell differentiation, proliferation, autophagy, and apoptosis.^[4–6]^ In recent years, intensive research has revealed the crucial roles of lncRNAs in the occurrence and progression of numerous diseases. For instance, a previous study reported that the levels of lncRNA DLEU1 elevated in colorectal cancer cases and that it promotes the malignant growth of cancer cells.^[7]^ Another study showed that high expression of lncRNA UCA1, which acts as a carcinogenic factor, is associated with poor prognosis of OA patients.^[8]^ The lncRNA SNHG5 has been shown to promote the proliferation and migration of chondrocytes through the miR-26a/SOX2 axis, suggesting its involvement in the progression of OA.^[9]^ The lncRNA plasmacytoma variant translocation 1 (PVT1) is located at chromosome 8q24.21 in humans. In recent years, lncRNA PVT1 has been extensively studied, as it is considered a cancer-promoting gene and a potential therapeutic target for various tumors.^[10,11]^ Studies in the past few years have demonstrated that PVT1 is upregulated in OA patients and can regulate chondrocyte apoptosis by acting as a sponge for miR-488-3p.^[12]^ A previous study showed that the cartilage tissue of OA patients exhibited elevated levels of PVT1 and that PVT1 inhibition can help reduce IL-1β-induced chondrocyte injury.^[13]^ These studies indicate that PVT1 can be used as a potential therapeutic target for OA patients. Therefore, further research is required to further elucidate the role of PVT1 in the progression of OA.

Herein, we assessed PVT1 expression in the articular cartilage tissues of OA and non-OA patients. Furthermore, through in vitro experiments, we analyzed the role and mechanism of action of PVT1 in OA progression.

## 2. Materials and methods

### 2.1. Clinical samples

We collected articular cartilage tissue samples from 24 OA and 24 non-OA patients who underwent total knee arthroplasty and internal fixation of tibial plateau fractures, respectively, in Yuebei People’s Hospital, China, from February 2016 to June 2018. OA patients were diagnosed using plain X-ray film and postoperative pathology. The postoperative pathology of non-OA patients was not suggestive of osteoarthritis and rheumatoid arthritis. Fresh articular cartilage tissue samples of the patients were stored at 80°C. There was no significant difference between the age, gender, weight, height, and other sociodemographic data of the two groups. All patients in the study provided signed informed consent. The study was carried out with the authorization of the Medical Ethics Committee of Yuebei People’s Hospital, affiliated with the Medical College of Shantou University. The study protocols were designed in accordance with the ethical tenets of the Declaration of Helsinki.

### 2.2. Cell processing

#### 2.2.1. Chondrocyte source and culture.

The ATDC5 chondrocytes were purchased from the ATCC cell bank. Cells were placed in Dulbecco’s Modified Eagle Medium (DMEM; Gibco) supplemented with 10% fetal bovine serum (FBS, Gibco). The medium was then cultured in an incubator under 5% CO_2_ at 37°C. Cells at the logarithmic growth stage were placed in 10 µg/L IL-1β medium containing 10 µg/L to simulate OA in vitro.

#### 2.2.2. Cell grouping and processing.

A pcDNA 3.1 plasmid was used as the vector to establish the PVT1 inhibitory plasmid (si-PVT1), the miR-497 over-expression plasmid (miR-497-mimics), and the AKT3 over-expression plasmid (sh-AKT3). Cells were transfected with si-NC was used as the control of for lncRNA, and miR-NC was used as the control of for miR-497. Subsequently, the plasmids were transfected into IL-1β-induced chondrocytes (Invitrogen™) and cultured for 24 hours.

### 2.3. Qrt-PCR

Total RNA was extracted from tissues and cells using the TRIzol kit (Invitrogen). UV spectrophotometry and agarose gel electrophoresis were used for the determination of RNA purity, concentration, and integrity. Then, 2 µg RNA was reverse transcribed into cDNA using a reverse transcription kit (Invitrogen), which, in turn, was amplified by PCR using PrimeScript RT Master Mix kit (Takara Bio, Tokyo, Japan). The PCR mixture comprised 10 µL SYBR qPCR mix, 0.8 µL upstream primers, 0.8 µL downstream primers, 2 µL cDNA, and 0.4 µL 50x ROX reference dye, and distilled water was added to the mixture to obtain a total volume of 20 µL. Conditions for the PCR reaction were as follows: 95°C for 60 seconds, 95°C for 30 seconds, and 60°C for 40 seconds (40 cycles). The 2^–△△ct^ was used for the quantification of qRT-PCR products.^[14]^

### 2.4. Western blot (WB)

Total protein was extracted using the RIPA lysis buffer (Thermo Scientific™, Waltham, MA). Bicinchoninic acid (BCA, Thermo Fisher™) assay was used for protein quantification. A 40 µg protein sample was extracted for 10% polyacrylamide gel electrophoresis (PAGE, 120 V). Then, the sample was transferred to a polyvinylidene difluoride (PVDF) membrane (Life Technologies, Gaithersburg, MD) and blocked with 5% skim milk for 2 hours at 37°C. The samples were then incubated with aggrecan (1:1500), collagen type II (1:1500), matrix metalloproteinase-9 (MMP-9, 1:1500), AKT3 (1:1500), and β-catenin primary antibody (1:1500) (Abcam, Cambridge, MA) overnight at 4°C. Following the removal of the primary antibody, the membrane was treated with horseradish peroxidase-labeled goat anti-rabbit secondary antibody (1:4000, Abcam) for 2 hours at 37°C. The membrane was then rinsed four times with PBS for 5 minutes each time. The electrochemiluminescence of the samples was measured in a dark room using the ECL method. Subsequently, grayscale analysis was conducted for the determination of the relative expression level of the target protein.

### 2.5. Cell proliferation

An MTT kit (Beijing Baiaolaibo Technology Co., Ltd., Haidian, Beijing, China) was used for the detection of cell metabolic activity. Cells were transferred to 96-well plates (density: 5000 cells/well), followed by the addition of 20 µL MTT solution into each well at different times for incubation at 37°C for 4 hours. Subsequently, the cells were incubated for 10 minutes with 150 µL dimethyl sulfoxide (DMSO). Finally, absorbance was assessed using a microplate reader at 490 nm wavelength.

### 2.6. Cell apoptosis

Cell digestion was performed with 0.25% trypsin, and the cells were configured into a 1 × 10^6^/mL suspension. Ten µL AnnexinV-FITC/PI (Shanghai Yeasen Biotechnology Co., Ltd., Pudong, Shanghai, China) was added successively to each well for incubation in a dark room at 37°C for 5 minutes. The samples were then subjected to flow cytometry (FCM) to assess the cellular apoptotic rate. BD FACSCanto™ II was used for flow cytometry.

### 2.7. Dual luciferase reporter assay

The starBase v3.0 and TargetScan 7.2 sites were used to identify potential target genes of PVT1 and miR-497. A Lipofectamine™ 2000 kit was used to establish PVT1-3′ UTR wild type (Wt), PVT1-3′UTR mutant (Mut), AKT3-3′UTR Wt, and AKT3-3′UTR Wt. Sections of RNA were transferred to the downstream of the luciferase reporter gene to sequence and identify the constructed plasmid, and then transfected to HEK 293T cells (ATCC) together with miR-497-mimics and miR-NC. Changes in luciferase activity were detected with the aid of a dual luciferase reporter assay kit (Solarbio, Bejing, China).

### 2.8. Statistical methods

All sample data were obtained from three separate experiments. SPSS18.0 software package (International Business Machines Corporation, Armonk, NY) was used for statistical analysis, while image rendering was done by GraphPad 7. Data were expressed in the form of mean ± standard deviation (mean ± SD). One-way analysis of variance (one-way ANOVA) and an independent sample’s *t* test were used for intergroup comparisons, an LSD *t* test was used for pairwise comparison, while repeated measurement ANOVA was utilized for expression at multiple time points. Bonferroni correction was used for back-testing. Statistical differences were indicated in all cases, where *P* < .05 indicated statistical significance.

## 3. Results

### 3.1. PVT1 is elevated in cartilage tissue of OA patients, and IL-1β-induced chondrocytes

**Figure 1:**
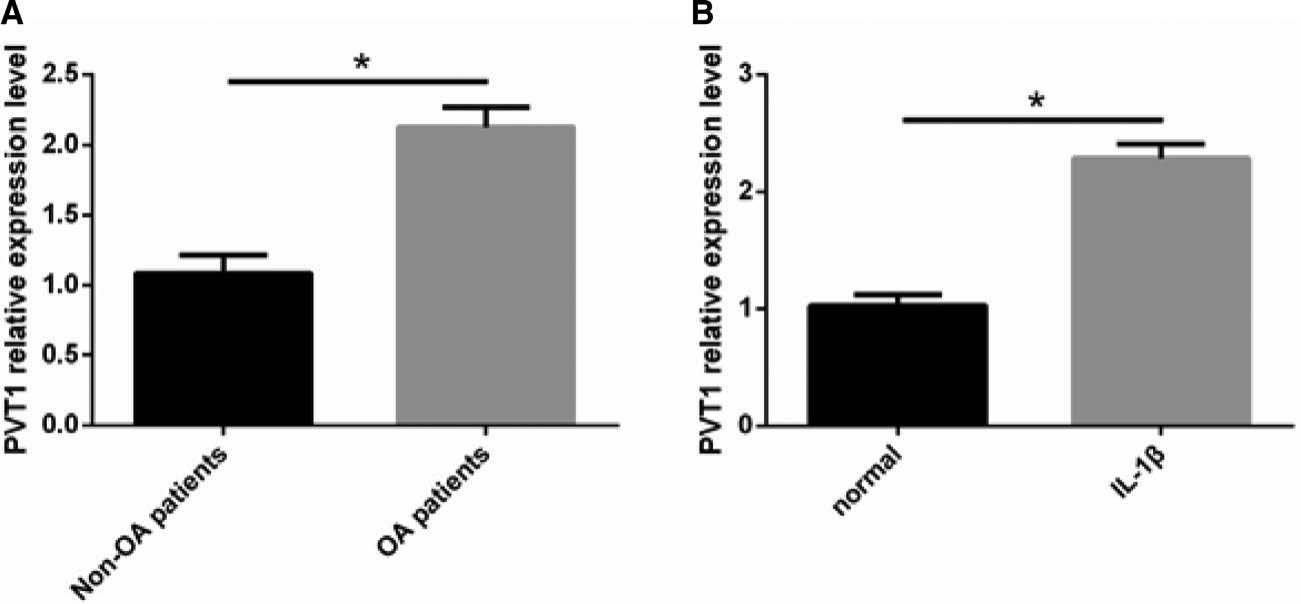
PVT1 is elevated in cartilage tissue of OA patients and IL-1β-induced chondrocytes. (A) qRT-PCR analysis revealed that PVT1 expression in the cartilage tissue of the OA group was significantly higher than that in the cartilage tissue of the non-OA group. (B) PVT1 expression was elevated in IL-1β-induced chondrocytes. Data are expressed as mean ± standard deviation (mean ± SD); n = 24; *P < .05. IL-1β = Interleukin-1 beta, OA = osteoarthritis, PVT1 = plasmacytoma variant translocation 1.

qRT-PCR analysis showed that PVT1 expression levels in the cartilage tissue of the OA patients were significantly higher than those in the cartilage tissue of the non-OA patients (Fig. 1A). Furthermore, PVT1 expression was elevated in IL-1β-induced chondrocytes (Fig. 1B).

### 3.2. PVT1 knockdown in IL-1β-induced chondrocytes enhances cell proliferation and decreases cell apoptosis

**Figure 2:**
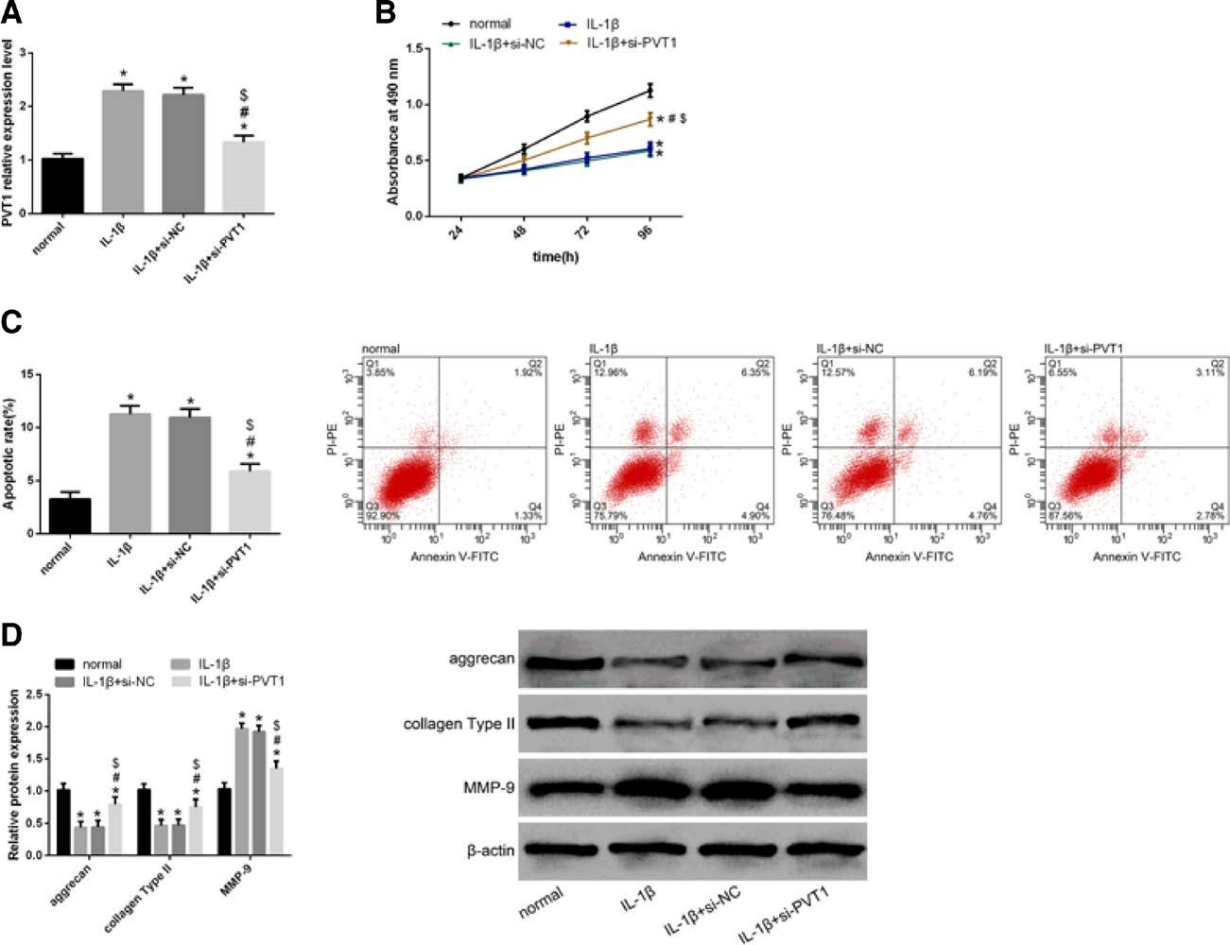
Effects of PVT1 on the proliferation, apoptosis, and injury of IL-1β-induced chondrocytes. (A) qRT-PCR analysis revealed that PVT1 expression was elevated in IL-1β-induced chondrocytes. PVT1 expression in IL-1β-induced chondrocytes reduced after si-PVT1 transfection. (B) MTT assay showed that IL-1β-induced chondrocytes exhibited reduced proliferation ability. However, after the transfection of si-PVT1 into IL-1β-induced chondrocytes, their proliferation ability was enhanced. (C) FCM revealed that IL-1β-induced chondrocytes exhibited increased apoptotic rates. However, their apoptotic rates decreased after si-PVT1 transfection. (D) WB analysis showed that IL-1β-induced chondrocytes exhibited downregulation of aggrecan and collagen type II and upregulation of MMP-9. Following the transfection of si-PVT1, the expressions of aggrecan and collagen type II increased, while MMP-9 expression decreased. Data are expressed as mean ± standard deviation (mean ± SD); n = 24; *P < .05 versus normal group; ^#^*P* < .05 versus IL-1β group; ^$^*P* < .05 versus IL-1β + si-NC group. IL-1β = Interleukin-1 beta, OA = osteoarthritis, PVT1 = plasmacytoma variant translocation 1.

Detection by qRT-PCR reveals that PVT1 expression is elevated in chondrocytes after IL-1β induction. Following the transfection of si-PVT1 into IL-1β-induced chondrocytes, PVT1 expression is reduced (Fig. 2A). Results from the MTT assay and flow cytometry showed that, compared to normal chondrocytes, IL-1β-induced chondrocytes exhibited decreased proliferation ability and enhanced apoptosis. However, PVT1 knockdown in IL-1β-induced chondrocytes led to a significant enhancement in cell proliferation (Fig. 2B). FCM shows that chondrocytes induced by IL-1β have increased cell apoptotic rates. Following the transfection of si-PVT1 into IL-1β-induced chondrocytes, cell apoptotic rate is decreased significantly (Fig. 2C). Furthermore, we also examined the expression levels of several extracellular matrix (ECM)-related proteins, including aggrecan, collagen type II, and MMP-9, in IL-1β-induced chondrocytes. Treatment with IL-1β led to upregulation of aggrecan and collagen type II and downregulation of MMP-9 (Fig. 2D). PVT1 knockdown reversed the effects of IL-1β induction of these genes in chondrocytes. This finding suggests that PVT1 might play a crucial role in OA progression.

### 3.3. Interplay of mir-497 and PVT1

**Figure 3:**
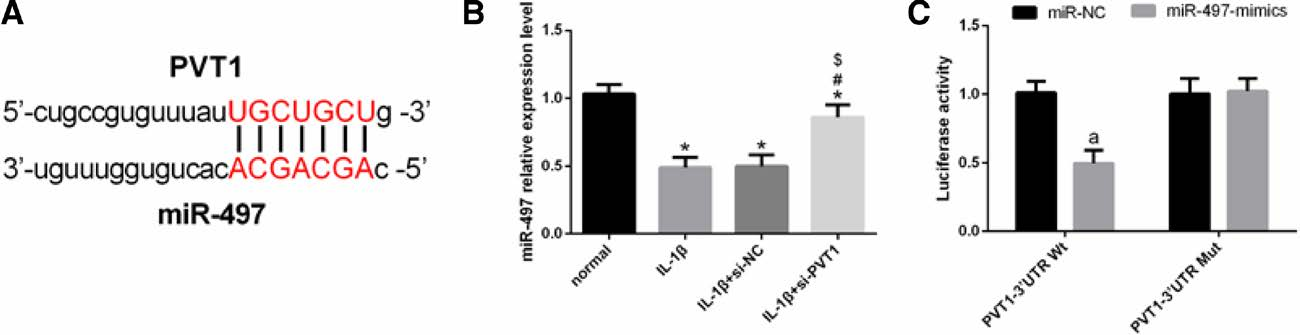
Interplay of miR-497 and PVT1. (A) There were targeted binding sites between miR-497 and PVT1. (B) qRT-PCR revealed that miR-497 expression decreased in chondrocytes after IL-1β-induction. However, miR-497 expression normalized after si-PVT1 transfection into IL-1β-induced chondrocytes. (C) Luciferase reporter assay showed that transfection of miR-497 mimics led to inhibition of the luciferase activity of PVT1-3′UTR Wt without affecting the luciferase activity of PVT1-3′UTR Mut. Data are expressed as mean ± standard deviation (mean ± SD); n = 24; *P < .05 versus normal group; ^#^*P* < .05 versus IL-1β group; ^$^*P* < .05 versus IL-1β + si-NC group; ^a^*P* < .05 versus miR-NC group. IL-1β = Interleukin-1 beta, PVT1 = plasmacytoma variant translocation 1.

We found targeted binding sites between miR-497 and PVT1 (Fig. 3A). We observed downregulation of the miR-497 expression in chondrocytes after IL-1β induction. However, miR-497 expression normalized after si-PVT1 transfection into IL-1β-induced chondrocytes (Fig. 3B). Subsequently, we explored the relationship between miR-497 and PVT1. We observed that miR-497 levels did not alter significantly post-PVT1 knockdown in IL-1β-induced chondrocytes, indicating that PVT1 knockdown leads to an increase in miR-497 levels. In addition, we further analyzed the relationship between miR-497 and PVT1 via a dual luciferase reporter assay. Our results showed that the transfection of miR-497 mimics led to inhibition of PVT1-3′UTR Wt luciferase activity without affecting the PVT1-3′UTR Mut luciferase activity, suggesting that miR-497 and PVT1 interacted with each other (Fig. 3C).

### 3.4. Mir-497 and PVT1 exhibit opposing functions

**Figure 4:**
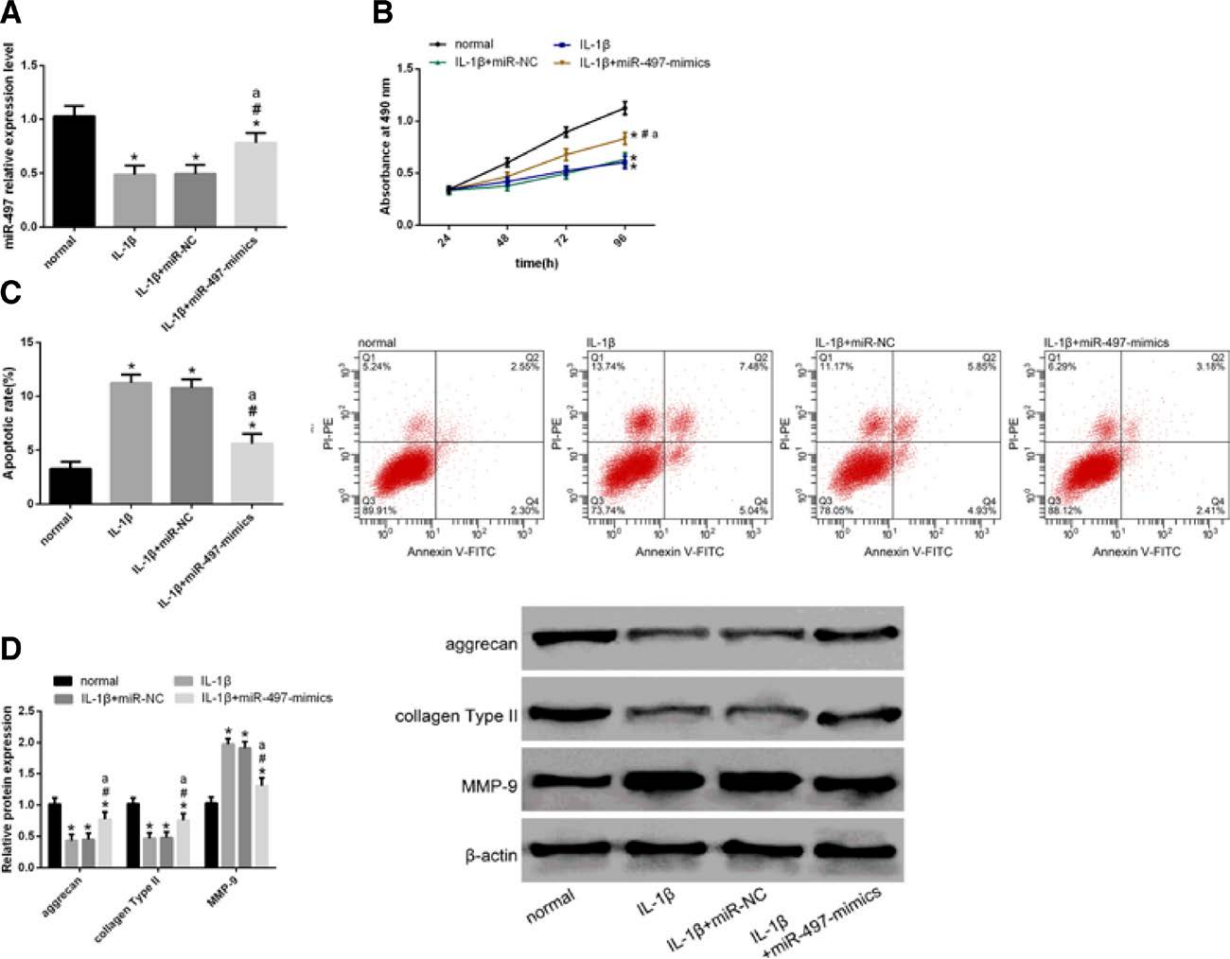
Function of miR-497 is the opposite of PVT1. (A) qRT-PCR revealed that miR-497 upregulation after transfection of miR-497-mimics into IL-1β-induced chondrocytes. (B) MTT assay showed that cell proliferation ability enhanced after transfection of miR-497 mimics into IL-1β-induced chondrocytes. (C) FCM showed that the cell apoptotic rate decreased after transfection of miR-497 mimics into IL-1β-induced chondrocytes. (D) WB analysis showed upregulation of aggrecan, and collagen type II and downregulation of MMP-9 after the transfection of miR-497 mimics into IL-1β-induced chondrocytes. Data are expressed as mean ± standard deviation (mean ± SD); n = 24; *P < .05 versus normal group; ^#^*P* < .05 versus IL-1β group; ^$^*P* < .05 versus IL-1β + si-NC group; ^a^*P* < .05 versus miR-NC group. IL-1β = Interleukin-1 beta, PVT1 = plasmacytoma variant translocation 1.

Next, we explored the role of miR-497 in the progression of OA. qRT-PCR revealed that miR-497 upregulation after transfection of miR-497-mimics into IL-1β-induced chondrocytes (Fig. 4A). To this end, we transfected mimics of miR-NC and miR-497 separately into IL-1β-induced chondrocytes. MTT assay showed that cell proliferation ability enhanced after transfection of miR-497 mimics into IL-1β-induced chondrocytes (Fig. 4B). FCM showed that the cell apoptotic rate decreased after transfection of miR-497 mimics into IL-1β-induced chondrocytes (Fig. 4C).

Moreover, WB analysis showed that these cells also exhibited upregulation of aggrecan and collagen type II and downregulation of MMP-9 (Fig. 4D). These results indicated that the function of miR-497 is opposite to that of PVT1.

### 3.5. Mir-497 mediates its function via negative regulation of AKT3

**Figure 5:**
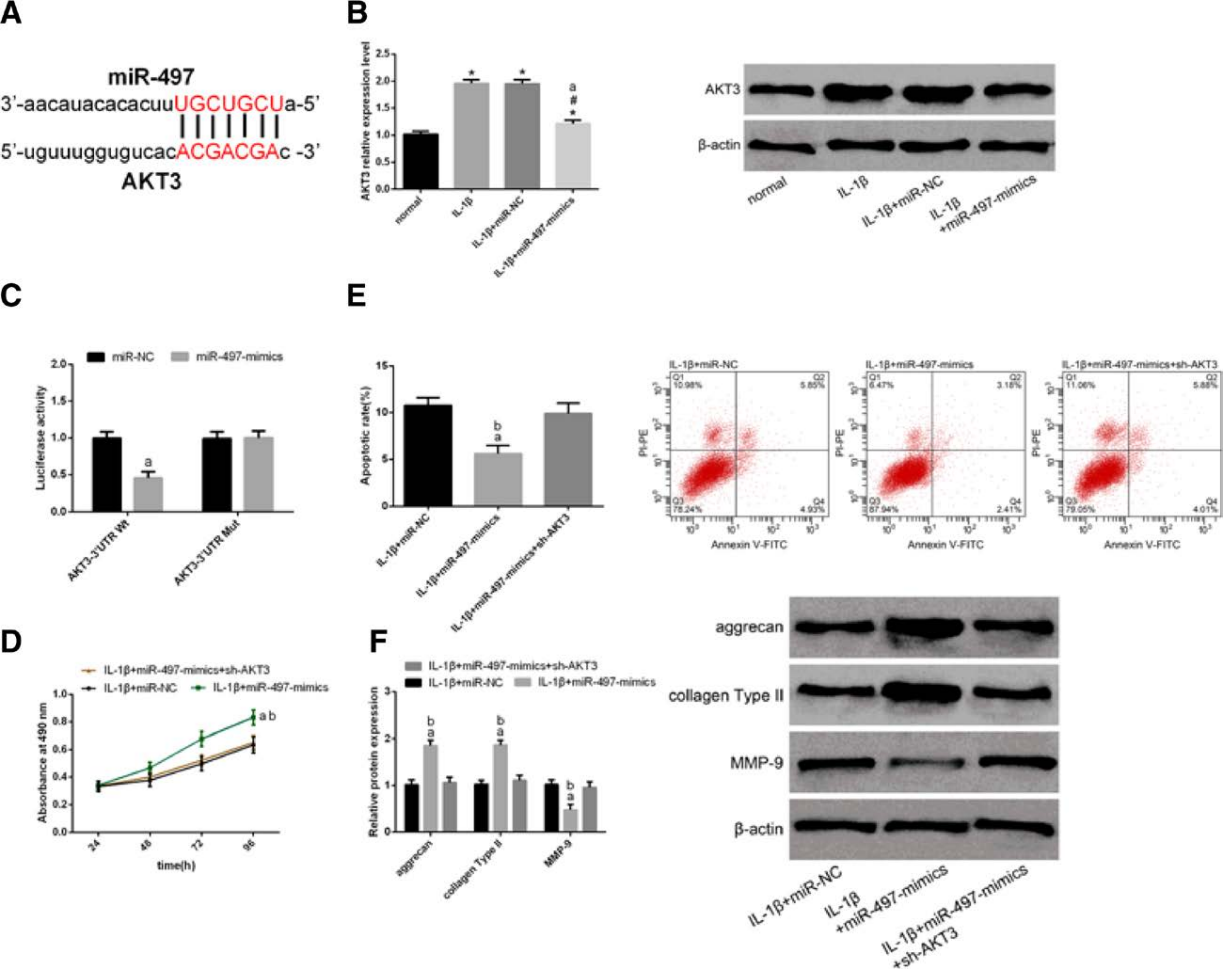
miR-497 acts through negative regulation of AKT3. (A) Binding sites of AKT3 and miR-497. (B) WB analysis revealed upregulation of AKT3 expression in chondrocytes after IL-1β induction. However, AKT3 expression decreased after transfection of miR-497 mimics into IL-1β-induced chondrocytes. (C) Luciferase reporter assay showed that miR-497 mimics inhibited the luciferase activity of AKT3-3′UTR Wt without causing significant changes in the luciferase activity of AKT3-3′UTR Mut. (D) FCM demonstrated that the proliferation ability of cells transfected with miR-497 mimics + sh-AKT3 was lower than that of cells transfected with miR-497 mimics. (E) Apoptotic rate of cells transfected with miR-497 mimics + sh-AKT3 was higher than that of cells transfected with miR-497 mimics. (F) WB analysis revealed that, compared with cells transfected with miR-497 mimics, cells transfected with miR-497 mimics + sh-AKT exhibited downregulation of aggrecan and collagen type II and upregulation of MMP-9. Data are expressed as mean ± standard deviation (mean ± SD); n = 24; *P < .05 versus normal group; ^#^*P* < .05 versus IL-1β group; ^a^*P* < .05 versus miR-NC group; ^b^*P* < .05 versus IL-1β + miR-497 mimics group. IL-1β = Interleukin-1 beta.

AKT 3 was identified as a potential target gene of miR-497 (Fig. 5A). WB analysis revealed upregulation of AKT3 expression in chondrocytes after IL-1β induction. However, AKT3 expression decreased after transfection of miR-497 mimics into IL-1β-induced chondrocytes. (Fig. 5B). Next, the association between miR-497 and AKT3 was analyzed by a dual luciferase reporter assay. miR-497 mimics were observed to inhibit AKT3-3′UTR Wt luciferase activity without inducing significant changes in AKT3-3′UTR Mut luciferase activity (Fig. 5C). Next, we transfected IL-1β-induced chondrocytes with miR-497-mimics and sh-AKT3. FCM demonstrated that the proliferation ability of cells transfected with miR-497 mimics + sh-AKT3 was lower than that of cells transfected with miR-497 mimics (Fig. 5D). Apoptotic rate of cells transfected with miR-497 mimics + sh-AKT3 was higher than that of cells transfected with miR-497 mimics (Fig. 5E). WB analysis revealed that, compared with cells transfected with miR-497 mimics, cells transfected with miR-497 mimics + sh-AKT exhibited downregulation of aggrecan and collagen type II and upregulation of MMP-9 (Fig. 5F). We observed that the effects of the miR-497 mimics on the proliferation, apoptosis, and expression of ECM-related proteins of OA chondrocytes were weakened by the presence of sh-AKT3, indicating that miR-497 acts via negative regulation of AKT3.

### 3.6. Regulation of PVT1 in chondrocytes depends on the modulation of the mir-497/AKT3 axis

**Figure 6:**
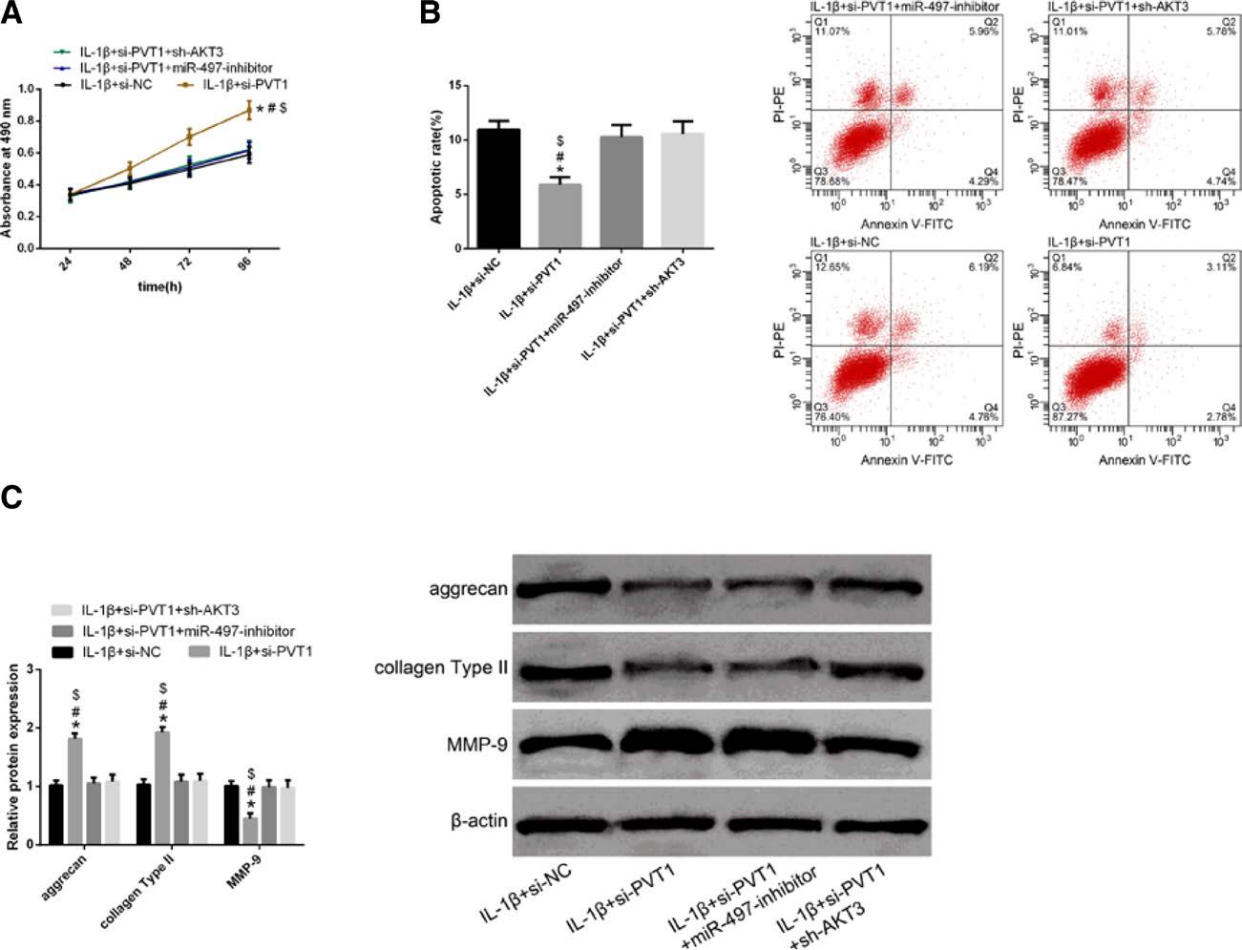
The regulation of PVT1 on chondrocytes depends on the regulation of the miR-497/AKT3 axis. (A) MTT assay indicated that transfection of either miR-497-inhibitor or sh-AKT3 reversed the effects of si-PVT1 transfection on the proliferation of IL-1β-induced chondrocytes. (B) FCM revealed that transfection of either miR-497 inhibitor or sh-AKT3 reversed the effects of si-PVT1 transfection on apoptotic rates of IL-1β-induced chondrocytes. (C) WB analysis revealed that transfection of either miR-497-inhibitor or sh-AKT3 reversed the effects of transfection of si-PVT1 on aggrecan, collagen, and MMP-9 expressions in IL-1β-induced chondrocytes. Data are expressed as mean ± standard deviation (mean ± SD); n = 24; *P < .05 versus IL-1β + si-NC group; ^#^*P* < .05 versus IL-1β + si-PVT1 + miR-497 inhibitor group; ^$^*P* < .05 versus IL-1β + si-PVT1 + sh-AKT3 group. IL-1β = Interleukin-1 beta, PVT1 = plasmacytoma variant translocation 1.

To further explore the mechanisms underlying PVT1 regulation by miR-497 or AKT3, we transfected a miR-497 inhibitor or sh-AKT3 into OA chondrocytes transfected with si-PVT1. We observed that both miR-497-inhibitor and sh-AKT3 reversed the effects of si-PVT1 on the proliferation (Fig. 6A), apoptosis (Fig. 6B), and expression of ECM-related proteins (Fig. 6C) of IL-1β-induced chondrocytes, suggesting that the regulation of PVT1 in chondrocytes depends on the regulation of the miR-497/AKT3 axis.

## 4. Discussion

To date, there is a lack of effective treatment methods for OA, which is a major cause of disability in the elderly.^[15]^ In recent years, with the advent of targeted drugs, identifying potential therapeutic targets of OA has become one of the important directions of contemporary OA research. Multiple factors are responsible for the incidence of OA. Among them, a significant factor is a decline in chondrocyte number.^[16]^ Various lncRNAs have been found to be involved in OA development via regulation of the behavior of chondrocytes. Hence, in recent OA research, lncRNAs have attracted a lot of attention.^[10,17]^ Previously, PVT1 levels have been shown to increase in chondrocytes of OA patients, suggesting that knocking down PVT1 in IL-1β-treated chondrocytes not only promotes cell activity and autophagy but also inhibits cell apoptosis and inflammatory response.^[17]^ In the current study, elevated levels of PVT1 were observed in the cartilage tissue of OA patients as well as IL-1β-induced chondrocytes, whereas PVT1 knockdown was found to promote proliferation and suppress apoptosis in these chondrocytes. Cartilage ECM plays a crucial role in maintaining cartilage structure and function.^[17]^ IL-1β inhibits the synthesis of anabolic genes (such as aggrecan and collagen type II) and enhances the levels of catabolic factors (such as MMP-9), thus intensifying the degradation of chondrocyte ECM.^[18,19]^ These findings corroborated the results of the present study, wherein we observed a downregulation of aggrecan and collagen type II and an upregulation of MMP-9 in IL-1β-induced chondrocytes. However, PVT1 knockdown alleviated the impact of IL-1β on the expression of these proteins. These results indicate that PVT1 knockdown could be considered a primary approach for OA treatment.

Growing evidence shows that lncRNAs can function as a molecular sponge of microRNA (miRNAs), thereby participating in a plethora of biological processes. Previous studies have shown that the lncRNA PVT1 promotes the proliferation and migration of pancreatic cancer cells by acting as a molecular sponge and regulating miR-448.^[20,21]^ Another lncRNA, MALAT1, promotes the malignant growth of triple-negative breast cancer cells via the regulation of miR-129-5p.^[22]^ Another study showed that PVT1 knockdown inhibits injury induced by IL-1β-induced chondrocytes by regulating the miR-27b-3P/TRAF3 axis.^[14]^ In the present study, using prediction software, we identified potential overlapping binding sites of PVT1 and miR-497 to investigate the potential mechanism of action of PVT1 in OA progression. We observed that PVT1 knockdown enhanced miR-497 levels in IL-1β-induced chondrocytes. The dual luciferase reporter assay also showed that miR-497 and PVT1 interacted with each other. miRNAs and lncRNAs are commonly found in the human body and are involved in the regulation of around a third of all human genes.^[23]^ It is noteworthy that the imbalance of miRNAs, which are key regulators of several genes, is considered to be a major cause of various diseases. Previous reports have shown that miR-497, which belongs to the miR-15/16/195/424/497 family, is downregulated in IL-1β-induced chondrocytes.^[24]^ These findings were in agreement with the results of the present study. In addition, the effects of IL-1β on chondrocyte proliferation and ECM were alleviated by upregulating miR-497 in IL-1β-treated chondrocytes, which indicated that both miR-497 and PVT1 could act as potential therapeutic targets for OA.

miRNAs target specific genes by inhibiting their mRNA and protein synthesis, thus preventing gene function.^[25]^ AKT3 protein belongs to the AKT serine/threonine kinase family and is known to participate in cell signaling pathways and processes, such as growth, proliferation, differentiation, and apoptosis.^[26,27]^ Its expression is elevated in many inflammatory diseases, including OA.^[28,29]^ Previous studies have revealed shown the AKT3 gene can be used as a target for various miRNAs, enabling the miRNAs to participate in the progression of several diseases. For instance, AKT3 has been shown to regulate the expression of miR-29a, which acts as a tumor suppressor in papillary thyroid carcinoma.^[30]^ AKT3 has also been shown to negatively regulate miR-384, which inhibits the proliferation of colorectal cancer cells.^[25]^ In the current study, we utilized prediction software to understand how miR-497 participates in OA progression. To this end, we identified the overlapping binding sites between miR-497 and AKT3. The expression of AKT3 was detected in chondrocytes transfected with miR-497-mimics and miR-NC, while the dual luciferase reporter assay revealed that miR-497 negatively regulated AKT3. Next, we transfected IL-1β-induced chondrocytes with miR-497 mimics and sh-AKT3. The impact of miR-497 mimics on the proliferation, apoptosis, and expression of ECM-related proteins in IL-1β-induced chondrocytes was reversed after sh-AKT3 transfection, suggesting that miR-497 acts via negative regulation of AKT3.^[25]^ Finally, we conducted rescue experiments to further investigate the mechanisms underlying PVT1 regulation by the miR-497/AKT3 axis. We observed that both the miR-497 inhibitor and sh-AKT3 reversed the effects of si-PVT1 on proliferation, apoptosis, and expression of ECM-related proteins of OA chondrocytes. These findings indicated that the regulation of PVT1 in chondrocytes is dependent on the regulation of the miR-497/AKT3 axis.

Although the present study confirmed that PVT1 knockdown could decrease the effects of IL-1β on proliferation and apoptosis of chondrocytes and degradation of chondrocyte ECM via the miR-497/AKT3 axis, it still has some limitations. Firstly, no animal experiments were conducted to analyze the effects of PVT1 on subchondral osteosclerosis and osteophyte formation. Secondly, neither the expression levels of miR-493 and AKT3 were measured in cartilage tissue of OA patients nor the clinical effects of PVT1, miR-497, and AKT3 were investigated in OA samples. Thirdly, potential mechanisms of action of PVT1 other than the miR-497/AKT3 axis were not explored. The above limitations are expected to be corrected by further experiments in follow-up studies.

## 5. Conclusion

In summary, PVT1 levels are elevated in the cartilage tissue of OA patients and IL-1β-induced chondrocytes. In addition, PVT1 knockdown alleviates the effects of IL-1β treatment on the proliferation and apoptosis of chondrocytes and ECM degradation in chondrocytes by regulating the miR-497/AKT3 axis.

## Author contributions

JX and XF performed the sampling, part of experimental research and data analysis, wrote and edited the manuscript. LQ performed part of experimental research and data analysis. XZ performed the study design, writing and editing of the manuscript. JX and QW contributed to critical review of the manuscript.

All authors read and approved the final manuscript and, therefore, had full access to all the data in the study and take responsibility for the integrity and security of the data.

**Conceptualization:** Jinming Xu.

**Data curation:** Jinming Xu, Xiang Fang, Ling Qin, Xinli Zhan.

**Formal analysis:** Jinming Xu, Xiang Fang, Ling Qin, Xinli Zhan.

**Funding acquisition:** Jinming Xu.

**Investigation:** Jinming Xu, Xiang Fang, Ling Qin, Xinli Zhan.

**Methodology:** Jinming Xu, Xiang Fang, Ling Qin, Qiang Wu, Xinli Zhan.

**Project administration:** Qiang Wu.

**Resources:** Qiang Wu.

**Supervision:** Qiang Wu.

**Validation:** Jinming Xu.

**Writing – original draft:** Jinming Xu.

**Writing – review & editing:** Jinming Xu, Qiang Wu, Xinli Zhan.
